# Solid-State NMR and Raman Spectroscopic Investigation of Fluoride-Substituted Apatites Obtained in Various Thermal Conditions

**DOI:** 10.3390/ma14226936

**Published:** 2021-11-16

**Authors:** Lukasz Pajchel, Leszek Borkowski

**Affiliations:** 1 Chair of Analytical Chemistry and Biomaterials, Department of Analytical Chemistry, Medical University of Warsaw, ul. Banacha 1, 02-097 Warsaw, Poland; 2 Chair and Department of Biochemistry and Biotechnology, Medical University of Lublin, Chodzki 1, 20-093 Lublin, Poland; leszek.borkowski@umlub.pl

**Keywords:** fluorapatite, hydroxyapatite, Raman, solid-state nuclear magnetic resonance, biomaterial

## Abstract

Fluoride-substituted apatites were synthesized by the standard sol-gel method and then calcined at three different temperatures: 800 °C, 1000 °C, and 1200 °C. Using a similar method, hydroxyapatite was synthesized and used as a reference material. The obtained powders were characterized by physicochemical methods: powder X-ray diffractometry, Raman spectroscopy, transmission electron microscopy, and solid-state nuclear magnetic resonance. All these methods allowed to identify additional α-TCP phase (tricalcium phosphate) in the HAP samples heated at 1000 °C and 1200 °C while fluoridated apatites turned out to be thermally stable. Moreover, Raman spectroscopy and NMR allowed to establish that the powders substituted with fluoride ions are not pure fluorapatite and contain OH- groups in the crystal structure. All the obtained materials had crystals with a shape similar to that of biological apatite.

## 1. Introduction

Hydroxyapatite (with the chemical formula Ca_10_(PO_4_)_6_(OH)_2_) (HAP) belongs to the group of calcium phosphates (CaPs) and has found wide application in the field of medicine due to its significant similarity to the mineral in bone tissue, enamel, dentin, and cement [1,2,3]. The biological apatite naturally present in tissue is not pure and stoichiometric HAP but is doped with numerous “foreign” ions. Na^+^, K^+^, Mg^2+^, and Sr^2+^ can partially replace Ca^2+^ ions; carbonates or hydrogen phosphates are frequently incorporated in place of orthophosphates; fluoride, chloride, and carbonate ions have taken their place in the channels of hydroxyl groups [1,2,4,5,6,7]. A special mineral component of enamel and dentin, and to a lesser extent of bone tissue, is an apatite doped with fluorine ions, called fluorapatite (FAP), with the chemical formula Ca_10_(PO_4_)_6_F_2_ [1,2,5,8,9].

Two basic trends can be distinguished in the creation of bone substitutes based on calcium phosphates (HAP in particular). The first one is based on synthesis of apatite with physicochemical properties similar to those of biological apatite. Apatites enriched with various ions are then formed, characterized by a nanocrystalline structure and a well-developed specific surface. However, it should be noted that such materials will undergo quite quick resorption [2,3,4].

The second direction is the development of materials that are much more resistant to resorption. HAP with such properties is similar in its properties to stoichiometric HAP. It is characterized by large crystals, a small specific surface area, and relatively few impurities. Interestingly, FAP can also be a compound characterized by high stability in the human body, so there is a growing interest in this material and the possibilities for its use in bone replacement therapy, dentistry, and oral implantology. It should be noted that both FAP and HAP are also used in conservative dentistry as components of toothpastes, and also in dental implantology as coatings, scaffolds, blocks, and cements [4,5,10].

As can clearly be seen, the morphology, chemical composition, crystallinity, and size of synthetic apatitic crystals and their aggregates play a crucial role in determining their properties and potential applications. First of all, the size of the crystals is very important. Nanosized HAP with high surface activity and an ultrafine structure similar to biological apatite is frequently used as a bone substitute. Previous studies have revealed that synthetic nanocrystalline HAP can more readily promote osteointegration and subsequent bone tissue formation. Therefore, it is believed that nanometric HAP bone is the best material to use for bone replacement and regeneration. Ceramic biomaterials based on HAP nanocrystals show significantly higher bioactivity and increased resorbability than micron-sized ceramics [3,4,11,12].

Stoichiometric HAP has a monoclinic structure *P*2_1_/*b*, whereas in nature, due to numerous substitutions with other ions, the hexagonal structure *P*6_3_/*m* appears. FAP has a hexagonal structure of *P*6_3_/*m*. The parameters of unit cells *a* and *c* are similar in both minerals and amount to *a* = 9.43 and *c* = 6.88 for HAP and *a* = 9.37 and *c* = 6.87 for FAP [4]. Parameters *a* and *c* are slightly smaller for FAP than HAP, which results from the larger radius of the hydroxyl ion in relation to the fluoride ion. It has also been observed that HAP has lower crystallinity compared to FAP [4].

In the literature, in addition to HAP and FAP, fluorine-substituted HAP (named fluorhydroxyapatites, FOHAP) is also discussed [5,10,13]. FOHAP is characterized, compared with HAP, by increasing in crystallinity, a decrease in crystal strain, and an increase in thermal and chemical stability. During the substitution of hydroxyal ions with fluoride ions inside the channels along the c axis of the crystal, both types of ions occur side by side. As a result of fluoride incorporation, covalent Ca-F bonds and/or hydrogen OH-F bonds are formed and the unit cell volume of apatite is reduced. F^−^ substitution of apatite increases its hardness and acid resistance (chemical durability) [10].

According to Tredwin et al., in order to obtain pure fluorapatite with the sol-gel method, fluoride should be added in an amount corresponding to the ratio P:F 3:1 [14]. Our research was focused on the synthesis of fluorapatite, therefore, here we investigated whether the addition of fluoride in the ratio of 3:1 to phosphorus in the synthesis process is sufficient to fully replace the hydroxyl groups of hydroxyapatite. Moreover, we investigated the physicochemical analysis of fluoridated apatite and HAP synthesized at various temperatures in order to compare their ultrastructure, chemical structure, and thermal stability. To achieve these goals, we combined powder x-ray diffractometry (PXRD), solid-state nuclear magnetic resonance spectroscopy (ssNMR) with Raman spectroscopy, and transmission electron microscopy (TEM). It should be noted that few works are available in the literature on the comparison of sintered FAP and HAP [9,15,16,17,18,19]. In [20], where both solid-state NMR and Raman spectroscopy were used to characterize FAP, researchers focused mainly on analyzing the effect of different fluor content on the structure and properties of the fluorhydroxyapatites (FOHAPs) and FAP obtained. What is more, we have not found any studies on combining NMR and Raman methods to investigate the properties of FAP and HAP sintered materials.

## 2. Materials and Methods

Fluoride-substituted apatite and HAP granules were synthesized in the laboratory of the Chair and Department of Biochemistry and Biotechnology (Medical University of Lublin, Poland), according to the procedure described in Polish Patent No. 235803 [21] and a previous article [22]. Both materials were synthesized by the sol-gel method. For synthesis, the following reagents were used: calcium hydroxide (Ca(OH)_2_; Acros Organics, Madrid, Spain), orthophosphoric acid (H_3_PO_4_; Chempur, Piekary Slaskie, Poland), and sodium fluoride (only for substituted apatite) (NaF; Chempur, Piekary Slaskie, Poland). The designated amount of NaF was dissolved in phosphoric acid solution to obtain a phosphorus–fluoride precursor solution, in which the P/F molar ratio was 3:1. This mixture was then added to a 10.8-fold amount of aqueous calcium hydroxide suspension (3.37 g Ca(OH)_2_ for every 100 mL H_2_O), with continuous stirring at room temperature (RT). The solution was allowed to age for 4 days at RT, and the resulting precipitate was washed several times with deionized water and was dried at 37 °C. HAP ceramics were made by the same method without the use of NaF. Finally, every precipitate was divided into three parts, and calcined at three different temperatures (800 °C, 1000 °C, or 1200 °C) for 2 h in a furnace. Crystallized HAP and fluoridated apatite materials were manually ground in a mortar to obtain granules of 0.2–0.3 mm. The materials obtained were named as follows:

HAP-800: hydroxyapatite granules sintered at 800 °C;
HAP-1000: hydroxyapatite granules sintered at 1000 °C;
HAP-1200: hydroxyapatite granules sintered at 1200 °C;
FAP-800: fluoride-substituted apatite granules sintered at 800 °C;
FAP-1000: fluoride-substituted apatite granules sintered at 1000 °C;
FAP-1200: fluoride-substituted apatite granules sintered at 1200 °C.

Powder X-ray diffraction (PXRD) analysis of the HAP and fluoride-substituted samples was performed using a Bruker D8 Advance diffractometer (Bruker, Karlsruhe, Germany). The measurements were carried out using CuKa radiation (λ = 1.54 Å) over the 2θ range of 10–80°, using a step size of 0.03°. For estimation of crystallite size, we calculated the values of full width at half maximum for the reflection of the (002) and (300) planes, representing the crystallites along the *c*-axis and *a*-axis, respectively. The Scherrer formula was used [23].

The sample morphology was determined using TEM (JEM 1400, JEOL, Tokyo, Japan). A drop of a sample suspension in ethanol was placed on a Cu grid covered with a formvar film, allowed to dry, and analyzed under the accelerating voltage of 80 kV. Average crystal size values were calculated from the data for 100 crystals, randomly chosen in the fields of representative view. 

Scanning electron microscope (SEM) images were taken using Nova NanoSEM 450 (FEI, Hillsboro, OR, USA) in low and high vacuum conditions.

The Raman spectroscopy measurements were performed using an iRaman 532 (B&W Tek, Newark, DE, USA) spectrometer. The conditions were as follows: 20× lens magnification, 200 repetitions of 1000 ms and 60% laser power, with a laser wavelength excitation of 532 nm. The spectra were recorded from 170 to 4000 cm^−^^1^ at RT.

The Fourier-transform infrared spectroscopy (FT-IR) spectroscopy mesurments were performed using a Spectrum 1000 (Perkin Elmer, Cleveland, OH, USA) spectrometer working at mid-infrared range. The conditions were as follows: 50 repetitions and resolution of 2 cm^−1^. The obtained samples were mixed with KBr in weight ratio 1:100 and pressed into pellets. The spectra were recorded from 400 to 4000 cm^−^^1^.

Raman and FT-IR spectra were processed using the GRAMS/AI 8.0 software (Thermo Scientific, Burlington, ON, USA, 2006). The processing included baseline correction, peak fittings, and second derivative.

The NMR spectra of ^31^P, ^19^F, and ^1^H nuclei were recorded under magic angel spinning MAS using a Bruker Avance III HD 600 spectrometer (Bruker, Karlsruhe, Germany)). For the ^31^P, ^19^F, and ^1^H MAS NMR experiments, the samples were spun at 7, 23, and 23 kHz, respectively. In the ^31^P experiments, conventional single pulse-acquire (Bloch decay, BD) and cross-polarization (CP, ^1^H→^31^P, ^19^F→^31^P) techniques were used. In the case of the ^1^H and ^19^F MAS NMR, only single pulse-acquire (Bloch decay) spectra were acquired. For all experiments, 32 scans were acquired. The peak fittings were done using the NutsPro (Acorn NMR, Livermore, CA, USA, 2007) computer programs. 

## 3. Results and Discussion

### 3.1. Powder X-ray Diffraction (PXRD)

**Figure 1:**
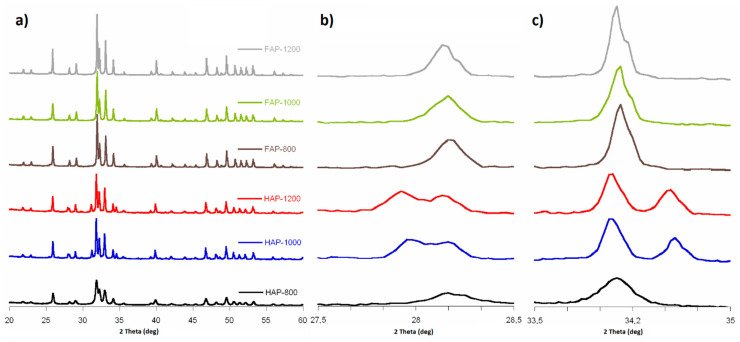
PXRD diffractograms: (**a**) 2Θ range from 20 to 60°; (**b**) 2Θ range from 27.5 to 28.5° (magnified 5×); and (**c**) 2Θ range from 33.5 to 35.0° (magnified 5×).

PXRD patterns of all the synthesized samples are shown in Figure 1a. The diffractograms showed main characteristic reflections from fluoridated apatites and HAP, and they were in great accordance with JCPDS:09-432 and JCPDs:15-876, respectively. All the expected reflections from HAP and FAP were present; they were conspicuous and well separated. The HAP-800 sample showed broader peaks then its HAP-1000 and HAP-1200 calcined forms.

Diffractograms of fluoridated apatites exhibited reflections originated only from the apatite structure, while in HAP PXRD patterns, we recorded additional peaks at 28.0°, 31.2°, and 34.6°. According to the literature and JCPDS:09-346 card, they may be assigned to the alpha-tricalcium phosphate (α-TCP) [6]. It should be noted that additional reflections of a crystalline phase of α-TCP were found in the HAP-1000 and HAP-1200 samples, which may have been caused by their calcination at a high temperature. The quantitative phase analysis based on Rietveld structure refinement showed 28.74% and 33.41% of the α-TCP phases in the HAP-1000 and HAP-1200 samples, respectively. What is interesting in the fluoride-substituted samples diffractograms is that there are no additional reflections. We may assume that the samples with incorporated fluoride ions were more thermally stable.

According to the HAP diffractogram peaks (310) and (222), their counterparts in samples with fluoride were displaced relative to HAPs, from 39.9° to 40.0° and from 36.7° to 36.8°, respectively. Shifts to the right side were caused by a decrease in the *a*-axis length of the hexagonal crystal lattice, induced by the lower ionic radius of F^−^ (133 pm) in comparison to that of OH- (140 pm) [20,24,25]. Calculated cell parameters (see Table 1) showed that lattice parameter *a* was smaller for substituted samples than for HAPs. Lattice parameter *c* was similar for all F-substituted and non-substituted samples. The calculated cell volume was bigger for HAP than for fluoridated samples, just as lattice parameter *c* was, due to the lower ionic radius of F^−^ ions. The crystallite size along the *c*-axis and *a*-axis was bigger for substituted samples, and in both groups, this grew with sintering. Larger F-substituted crystallites were caused by an increase in crystallinity by the incorporation of fluoride into the HAP lattice. The increased size of crystallites with heating was probably due to their sintering.

### 3.2. Transmission Electron Microscopy (TEM)

**Figure 2:**
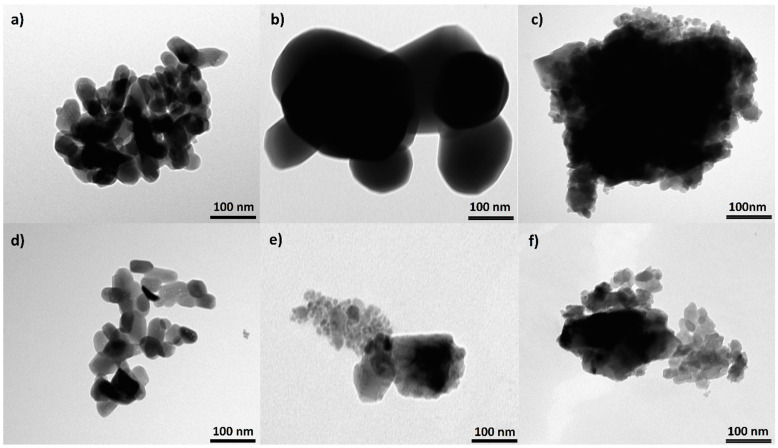
TEM pictures of (**a**) HAP-800, (**b**) HAP-1000, (**c**) HAP-1200, (**d**) FAP-800, (**e**) FAP-1000, and (**f**) FAP-1200.

Images taken using the TEM method are presented in Figure 2. The representative image of a HAP sample shows plate-like crystals growing in size with the heating temperature. The HAP-800 crystals were similar to natural bone crystals (see Table 2), and were 30–50 nm and 15–30 nm wide and 2–10 nm thick [3]. Crystals in HAP-1000 samples were five times bigger, but all of them still had a plate-like morphology. For the HAP-1200 sample, we observed two types of crystals: plate-like crystals two times smaller than in the HAP-800 sample and very large with sharp edges. TEM images confirmed the earlier suggestion of two phases of HAP and α-TCP in HAP-1200 samples. The FAP-800 (Table 2) crystals were a bit larger than HAP-800 while maintaining a plate like shape. There was a noticeable greater increase in length than in width compared to the HAP-800. The FAP-1000 and FAP-1200 have two types of crystals: smaller and much larger. Large crystals can be agglomerates or solid crystals, but this cannot be observed by the TEM method.

### 3.3. Scaning Electron Microscopy (SEM)

**Figure 3:**
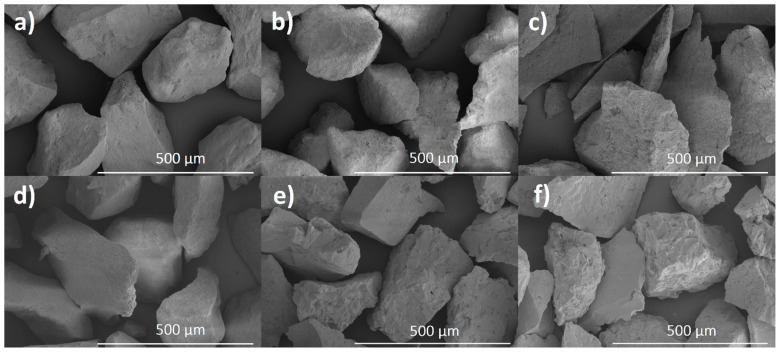
SEM pictures of (**a**) HAP-800, (**b**) HAP-1000, (**c**) HAP-1200, (**d**) FAP-800, (**e**) FAP-1000, and (**f**) FAP-1200.

Pictures of the samples acquired by SEM method are presented in Figure 3. The images show particles about 20 microns in size. From previous PXRD and TEM studies, it is known that the individual crystals in the samples are less than 100 nanometers in size, so these particles are probably agglomerates. The surface of HAP-800 and FAP-800 samples was smooth, while the other samples showed surface unevenness which was the largest for the sample sintered at 1200 °C. Few pores were also visible in the HAP-1200 and FAP-1200 samples.

### 3.4. Raman Spectroscopy

**Figure 4:**
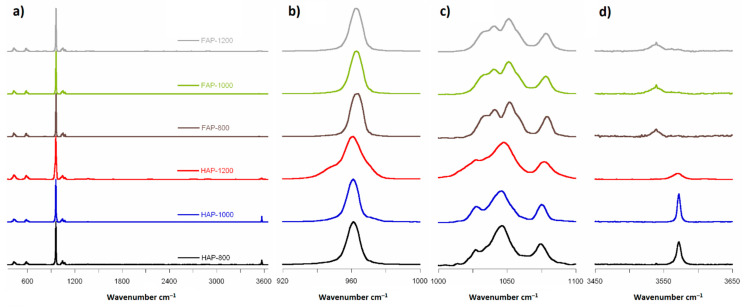
Raman spectra: (**a**) range 350 to 3600 cm^−^^1^; (**b**) range 920 to 1000 cm^−1^; (**c**) range 1000 to 1100 cm^−1^ (magnified 10×); and (**d**) range 3450 to 3650 cm^−1^ (magnified 20× for FAPs and 5× for HAPs).

The spectra obtained by Raman spectroscopy for all synthesized samples are shown in Figure 4a. The Raman spectra analysis showed the same main groups of bands typical for HAP and FAP spectra. All spectra had the strongest PO_4_^3−^ band at about 960 cm^−1^, two regions with a few bands at 1000–1100 cm^−1^ and 400–650 cm^−1^, and one weak band at range 3500–3600 cm^−1^ of OH groups.

For plotting, we normalized all spectra to the strongest band of spectra from ν_1_PO_4_, recorded at 961 cm^−1^ for HAP and 964 cm^−1^ for substituted material. In fluoride-containing samples, the PO_4_^3−^ band associated with the P–O stretch shifted upfield, which suggested a shortening of the P–O bond due to the content of fluorine ions. Chen et al. [20] have suggested that replacement of OH^−^ ions by smaller F^−^ ions increases the electrostatic attraction between the oxygen atoms in the phosphate tetrahedra, which then produces a shortening of the P–O bonds and an increase in vibrational frequency [20]. The full width at half-height (FWHH) of the phosphate symmetric band stretched at 960 cm^−1^, decreasing with increasing heating temperature for HAP-800 and HAP-1000 samples, and increasing with heating temperature for FAP samples. The FWHH parameter has also been used in other studies [20,26] to measure crystallinity of HAP samples using the Raman spectroscopy method. Increased crystallinity caused a decrease in the FWHH of the band. We observed decreased crystallinity with increasing heating temperature for HAP-800 and HAP-1000 samples, and increasing crystallinity with increasing heating temperature for FAP samples. What is more, in the case of HAP-1200 samples, we found a curve-fitting peak for two bands (Figure S1 in Supplementary Materials): the main at 961 cm^−1^ assigned from apatite structure and a very weak band which can be assigned to PO_4_^3−^ from α-TCP.

For the next region (from 1000 to 1100 cm^−1^), in the available literature [13], seven bands have been reported: at 1030, 1034, 1040, 1048, 1056, 1063, and 1077 cm^−1^. In the HAP samples, we observed only six in this region (at 1028, 1034, 1047, 1057, 1063, and 1075 cm^−1^) and five in the case of fluoridated samples (at 1033, 1043, 1053, 1063, and 1078 cm^−1^). Pennel et al. [13] suggest only four bands for pure fluorapatite: at 1034 (very weak), 1042, 1053, and 1081 cm^−1^, with an exclusion band at 1063 cm^−1^. Finding five bands for FAPs may suggest incomplete substitution of OH^−^ ions by F^−^ and formation of FOHAP [13].

The Raman region from 3500 to 3600 cm^−1^ exhibited one band: at approximately 3573 cm^−1^ and 3537 cm^−1^ for HAPs and fluoridated apatites, respectively (see Figure 4d). The band at 3573 cm^−1^ came from the structural hydroxyl groups occurring in the channels along the *c*-axis of the HAP crystals. It should be noted that the relative intensity of this band decreased with increasing temperature of calcination. This may be another confirmation of the thermal decomposition of HAP and the loss of structural hydroxy groups. Interestingly, in the F-substituted material spectra, an easily detectable band at about 3537 cm^−1^ occurred. According to literature [20] and structural data, there should be no bands in this area. Therefore, we can assume that this band came from OH groups, caused by incomplete substitution of F^−^ ions, and that the samples were not pure FAP. It is likely that places with fluoride ions in the channels on the lateral edge of the cell along the *c*-axis were also occupied by OH groups. Bands 3537 cm^−1^ of the OH groups tended to be for FOHAP [13].

The 311 cm^−1^ bands (Ca-F) reported by Penel et al. [13] in Raman spectra were not seen in our FAP samples, and this is probably due to incomplete F^−^ ion substitution.

The next finding was the same band for all samples at 430 cm^−1^ and 450 cm^−1^ from ν_2_PO_4_ (Figure S2a in Supplementary Materials). The next group of ν_4_PO_4_ contained four bands: at 578 cm^−1^, 587 cm^−1^, 606 cm^−1^, and 616 cm^−1^ (Figure S2b in Supplementary Materials). Like Penel et al. [13], we did not detect (for any sample) a band from the hydroxyl liberation mode at 630 or 655 cm^−1^. A literature review of Raman band positions at HAP and FAP is available in Supplementary Materials in Table S1.

### 3.5. Fourier Transmition Infrared Spectroscopy (FT-IR)

**Figure 5:**
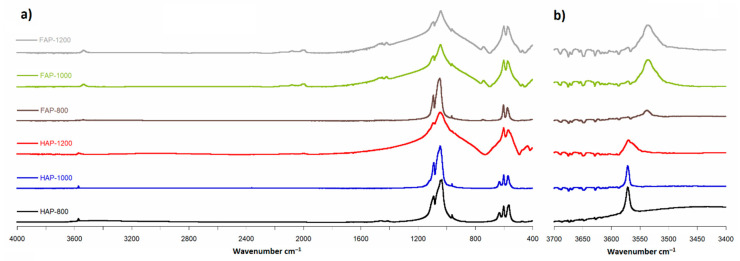
FT-IR spectra: (**a**) range 400 to 4000 cm^−1^; (**b**) range 3400 to 3700 cm^−1^ (magnified 10×).

The FT-IR spectra obtained for all samples are presented in Figure 5. In order to confirm the position of the signals, we counted the second derivative (Figure S3 in Supplementary Materials).

The FT-IR spectra analysis showed the same main groups of bands typical for HAP and FAP spectra. All spectra had the strongest PO_4_^3−^ band at about 1036 cm^−1^, region with a few bands at 500–650 cm^−1^ and one weak band at range 3500–3600 cm^−1^ of OH groups. Figure 5b shows the characteristic for FOHAP shift of the signal from the OH groups from 3571 cm^−1^ to 3536 cm^−1^ [13,27].

### 3.6. Solid-State Nuclear Magnetic Resonance Spectroscopy (ssNMR)

**Figure 6:**
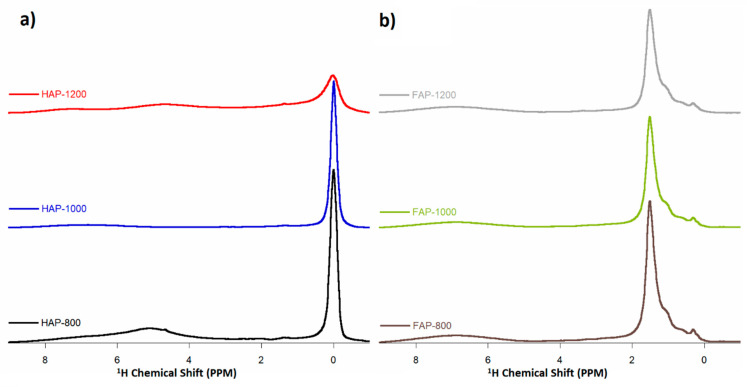
^1^H MAS NMR spectra of (**a**) HAP samples and (**b**) F-substituted apatite samples (multiplied 5×).

**Figure 7:**
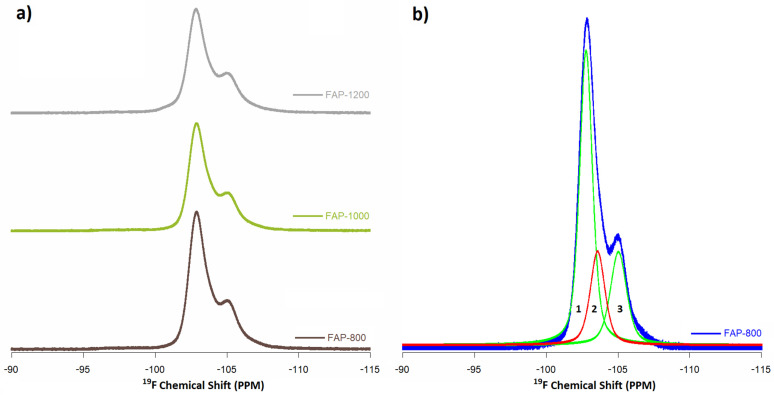
^19^F MAS NMR spectra of (**a**) FAP samples and (**b**) curve fitting of FAP-800 samples (1. crystalline phase, 2. apatite-like boundary layer from FOHAP, 3. amorphous phase).

**Figure 8:**
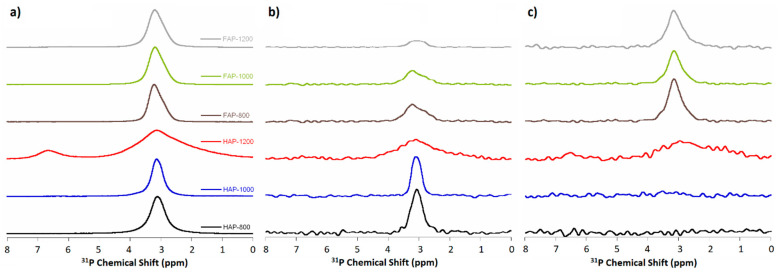
NMR spectra of (**a**) ^31^P BD MAS NMR; (**b**) ^1^H→^31^P CP MAS NMR; and (**c**) ^19^F→^31^P CP MAS NMR.

The ssNMR spectra made for the test samples are presented in Figure 6, Figure 7 and Figure 8. The ^1^H MAS NMR spectra of HAPs and fluoride-containing samples are shown in Figure 6. The NMR spectra were normalized to the intensity of ^31^P MAS NMR spectra to offset the different numbers of samples tested in the rotor. This made it possible to compare the obvious intensity of the signals for the samples. For HAP-800, we observed two main signals at 0.0 ppm, corresponding to structural OH grups, and another at 5.0 ppm, corresponding to water molecules adsorbed on the surface of the powder [3,20]. It should be noted that the structural hydroxyl groups are located at the edges of unit cells in the—O–H O–H O–H O–H—columns, parallel to the *c*-axis. The oxygen atoms are too distant (0.344 nm) from one another to form hydrogen bonds, so the ^1^H NMR signal of the structural OH groups from apatites appeared at 0 ppm [28]. In the spectra of HAP-1000 and HAP-1200 samples, the signal from surface water disappeared, which was related to their efficient heating. On the other hand, the signal from the hydroxyl groups weakened with increasing heating temperature, which was probably caused by the loss of hydroxyl groups during this process and decomposition of HAP. HAP can decompose at high temperatures into α-TCP and calcium oxide, according to the reaction in the equation below [29].

Ca_10_(PO_4_)_6_(OH)_2_ → 3 Ca_3_(PO_4_)_2_ + CaO + H_2_O

As expected, in the fluoridated samples spectra, no signal from hydroxyl groups was observed at 0.0 ppm, while three different signals were observed: stronger at 1.5 ppm, weaker at 1.0 ppm, and a weak blur at 7.0 ppm. The 1.5 ppm line probably originated from protons from OH^−^ groups near F^−^ ions entering the columns [7,30]. Ren et al. [7] suggested that the 0.0 ppm signal in HAP is assigned to the OH group, carrying the—O–H O–H O–H O–H—motif in the HAP structure [7]. When OH^−^ is replaced with F^−^ ions in the FAP, an—O–H O–H O–H F—motif is formed, resulting in a 1.5 ppm signal. It has also been observed in HAP samples that as the heating temperature increases, the 1.5 ppm signal decreases. The presence of this signal (and its loss at higher temperatures) confirmed that the obtained fluoride-substituted apatites were FOHAP. OH ions remained in them and lowered with increasing temperature. In turn, the broad signal (about 7 ppm) was from surface HPO_4_^2−^ [31]. Owing to the disorder in the surface layer, this ^1^H line shape was extremely broad.

^19^F MAS NMR spectra for fluoridated samples were recorded at a spinning frequency of 23 kHz, which allowed two signals (Figure 7, normalized to the intensity of ^13^P MAS NMR spectra) to be observed and curve fitting to three signals: −103 ppm, −104 ppm, and −105 ppm (Figure 7b).

A signal at −103 ppm has previously been recorded [7,30,32] for fluorapatite in the fluorapatite crystalline domain. Vyalikh et al. [32] proved assignment of these to pure crystalline fluorapatite. A position of −104 ppm points to an apatite-like boundary layer or should be from FOHAP, indicating that the local apatite structure around this fluorine site is preserved. This confirmed that the apatites synthesized with fluoride (according to a ratio of P:F 3: 1) were FOHAP (already spotted by PXRD and Raman spectroscopy). Moreover, −105 ppm signals further away from the main signal at −103 ppm were assigned an amorphous phase, composed of Ca_3_F or for CaF_2_ [7,32]. A decrease in the intensity of this complex signal was also observed with an increase in heating temperature.

^31^P MAS NMR spectra were recorded by three different experiments: the conventional single pulse-acquire (Bloch decay, BD) and two cross-polarization experiments: from protons to phosphorus-31 nuclei and from fluorine-19 to phoshorus-31 nuclei (CP, ^1^H→^31^P, ^19^F→^31^P). All the spectra showed the main signal at approximately 3 ppm.

The ^31^P BD experiment (Figure 8a) showed the total content and distribution of phosphorus-31 nuclei. All spectra (except HAP-1200) contained one signal with a position of 3.1 ppm for HAPs and 3.5 ppm for fluoridated samples. The signal at approximately 3.1 ppm was characteristic for pure, unsubstituted HAP. In turn, the signal at 3.5 ppm (present in F-substituted apatites spectra) was composed of two lines: at 3.1 ppm and at 3.5 ppm. We assumed that the signal at 3.5 ppm may be the result of the presence of F^−^ ions in the channel along *c*-axis (see Figure S4 in Supplementary Materials).

The appearance of an additional signal at 6.7 ppm for HAP-1200 confirmed previously observed decomposition of the sample and formation of the second phase (α-TCP). This line was very wide, which suggests a large disorder of the structure and the appearance of non-apatite phosphate.

The FWHH of the phosphate signal at about 3.1 ppm decreased with increasing heating temperature for HAP-800 and HAP-1000 samples, and increased with increasing heating temperature for fluoride-containing samples. The FWHH parameter has been used in studies reported in the literature [20,26] to measure the crystallinity of HAP samples using the NMR spectroscopy method. Increased numbers of crystallines cause a decrease in the FWHH of the signal. The FWHH of the phosphate signal at about 3.1 ppm decreased with increasing heating temperature for HAP-800 and HAP-1000 samples, and increased with increasing heating temperature for F-substituted samples (as with the 960 cm^−1^ band in Raman spectra).

In the cross-polarization (CP and 1H→31P) experiment, the spectra (shown in Figure 8b) revealed phosphorus nuclei with protons in their environment.

The CP and ^1^H→^31^P (normalized to the intensity of ^13^P MAS NMR spectra) experiment (for all samples) registered a decreasing signal with increasing heating temperature for both simple types and was much weaker for fluoridated apatite signals than for HAPs. This was due to the lower intensity of the OH groups in substituted apatites and their loss as the temperature increased. These experiments unequivocally confirmed the FOHAP nature of synthesized fluoride-substituted apatite materials.

The cross-polarization (CP, ^19^F→^31^P) experiment (shown in Figure 8c) revealed phosphorus nuclei with fluorides in their environment.

The CP, ^19^F→^31^P (normalized to the intensity of ^13^P MAS NMR spectra) experiments (for all samples) showed no signal for HAPs, as expected, and a single signal for all F-substituted apatites, decreasing with increasing heating temperature.

## 4. Conclusions

In this study, we synthesized two types of materials and calcined them at three different temperatures. The results obtained from PXRD, TEM, Raman, and solid-state NMR spectroscopy revealed that the materials obtained were hydroxyapatite and fluorhydroxyapatites.

It turned out that the addition of fluoride in the ratio of P:F 3:1 during synthesis process did not lead to the synthesis of pure FAP but rather FOHAPs, and contained additional OH groups in the channel along the c-axis.

Physicochemical tests on HAP-1200 samples confirmed the presence of two phases: apatite and α-TCP, which is characteristic of samples heated at a high temperature. Interestingly, α-TCP was not formed in F-containing samples, so it can be concluded that fluoride ions stabilized the structure of apatite.

It has been proven that heating causes loss of OH groups in both HAP and fluoridated samples. On the other hand, the crystallinity increased for HAP and decreased for F-substituted apatite with heating temperature.

Our work showed the usefulness of the methods ssNMR and Raman spectroscopy used for a detailed structural analysis of the obtained calcium phosphates.

The next work will be devoted to the biological properties and potential use of this materials in orthopedics and tissue engineering.

## Figures and Tables

**Table 1:** Crystal parameters obtained for each sample based on the PXRD diffractograms.

Sample	a (Å)	c (Å)	Cell Volume (Å^3^)	Crystallite Size Along c-Axis	Crystallite Size Along a-Axis
FAP-800	9.379 (3)	6.885 (2)	524.6 (3)	58 ± 4 nm	49 ± 4 nm
FAP-1000	9.376 (2)	6.885 (3)	524.2 (3)	54 ± 3 nm	47 ± 3 nm
FAP-1200	9.377 (1)	6.887 (3)	524.5 (2)	74 ± 4 nm	68 ± 4 nm
HAP-800	9.415 (2)	6.881 (1)	528.2 (1)	30 ± 2 nm	24 ± 2 nm
HAP-1000	9.419 (3)	6.882 (3)	528.8 (3)	55 ± 4 nm	44 ± 3 nm
HAP-1200	9.408 (2)	6.885 (2)	527.7 (1)	54 ± 3 nm	48 ± 3 nm

**Table 2:** Mean crystal size (nm) of HAPs and FAPs samples calculated by TEM.

Material	Type of Crystals	Width	Length
HAP-800	uniform	32 ± 9	49 ± 19
HAP-1000	uniform	154 ± 75	205 ± 122
HAP-1200	small crystals	15 ± 6	22 ± 11
	sharp edges crystals	166 ± 124	269 ± 179
FAP-800	uniform	46 ± 35	70 ± 46
FAP-1000	small crystals	21 ± 13	32 ± 19
	sharp edges crystals	121 ± 51	212 ± 52
FAP-1200	small crystals	22 ± 12	33 ± 20
	sharp edges crystals	163 ± 56	264 ± 88

## Supplementary Materials

The following are available online at https://www.mdpi.com/article/10.3390/ma14226936/s1. Figure S1: Raman spectra of curve fitting HAP-1200 range 900 cm^−1^ and 1000 cm^−1^, Figure S2: Raman spectra (**a**) range 400 cm^−1^ and 475 cm^−1^ (**b**) range 550 cm^−1^ and 650 cm^−1^, Figure S3: FT-IR spectra with the corresponding second derivative, Figure S4: ^19^F MAS NMR spectra of curve fitting FAP-800 sample, Table S1: Raman vibration modes of phosphate and hydroxyl in the apatitic samples.
