# Supplementary material for: Solid-State NMR and Raman Spectroscopic Investigation of Fluoride-Substituted Apatites Obtained in Various Thermal Conditions
Source: Materials (Basel). 2021 Nov 16;14(22):6936. doi: 10.3390/ma14226936 (PMC8624616; doi:10.3390/ma14226936)
Supplement: Supplementary file 1 [file materials-14-06936-s001.zip › materials-1449513-supplementary.pdf]

Supplementary materials

article title: **Solid-state NMR and Raman spectroscopic investigation of fluoride-substituted apatites obtained in various thermal conditions**

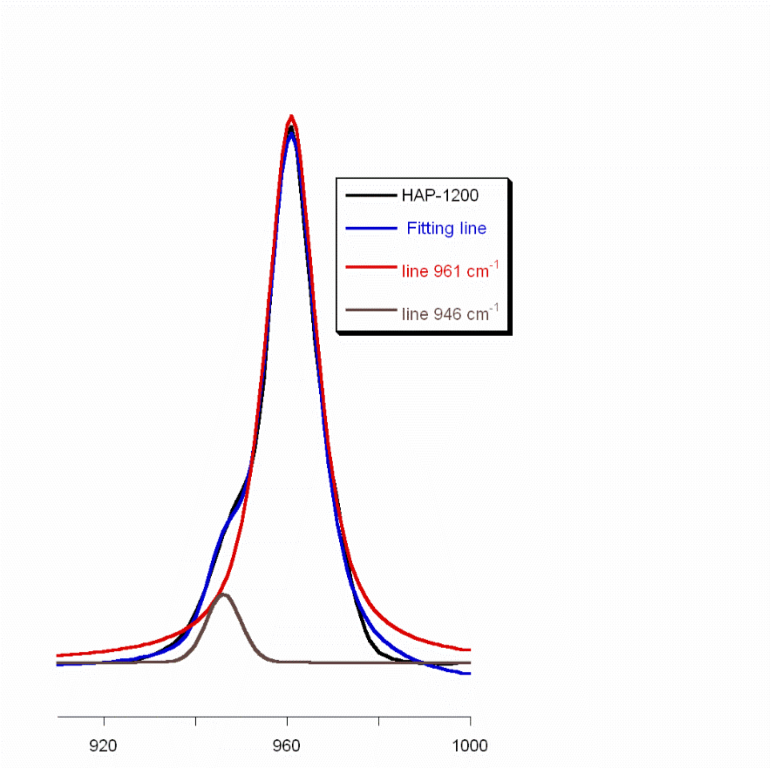

Figure S1. Raman spectra of curve fitting HAP-1200 range 900 cm^{-1} and 1000 cm^{-1}.

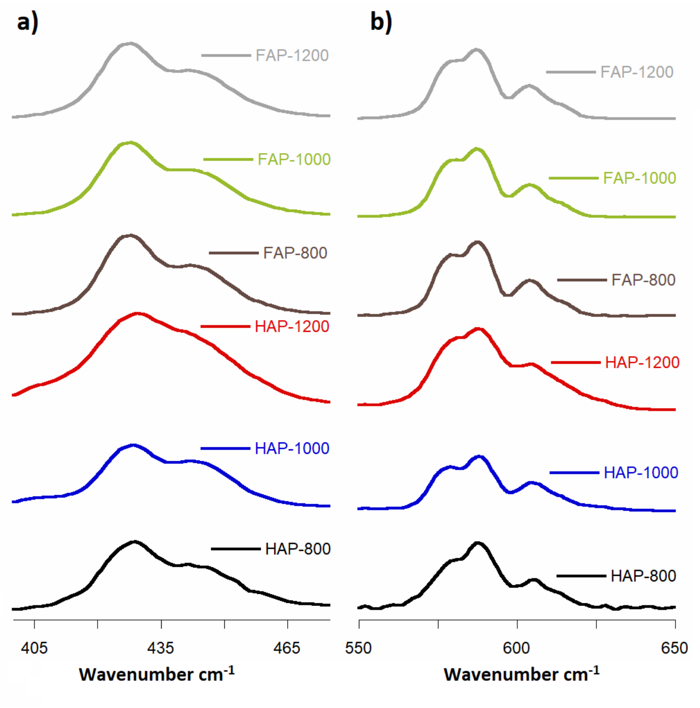

Figure S2. Raman spectra a) range 400 cm^{-1} and 475 cm^{-1} b)) range 550 cm^{-1} and 650 cm^{-1}

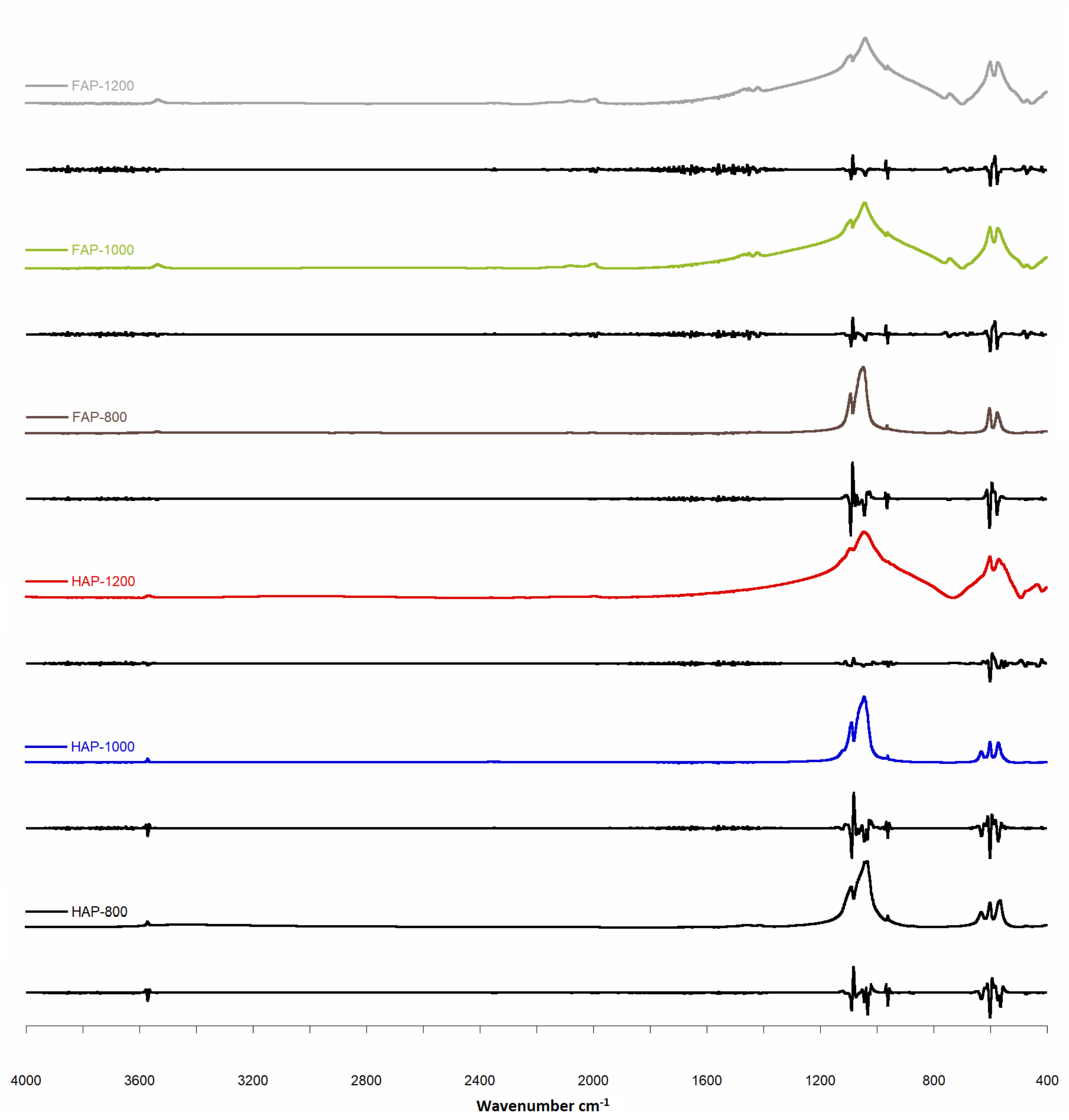

Figure S3. FT-IR spectra with the corresponding second derivative.

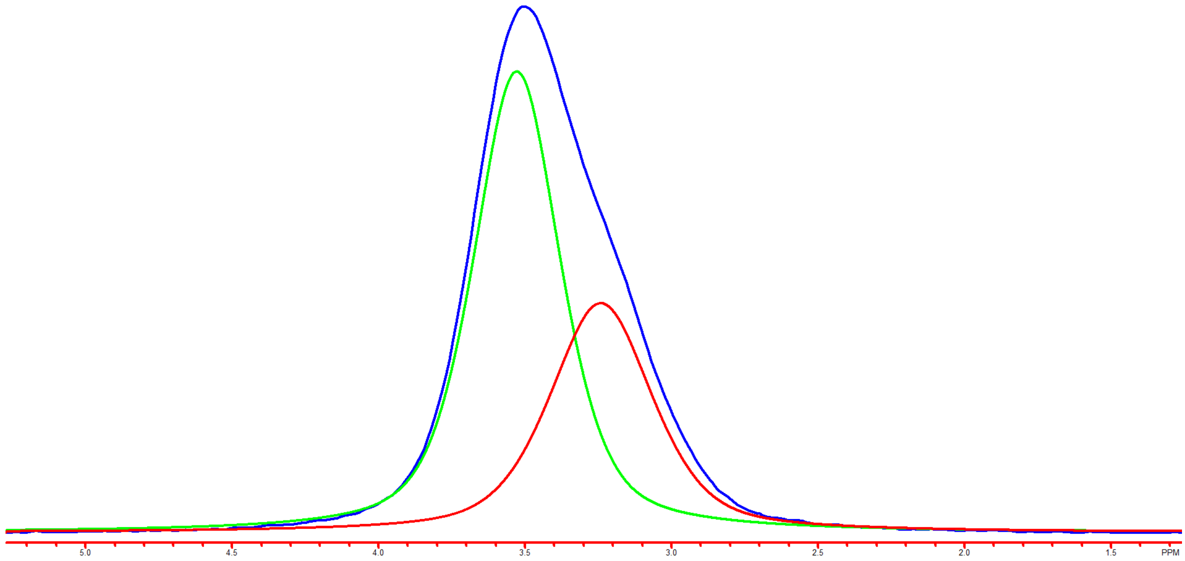

Figure S4. ^{19}F MAS NMR spectra of curve fitting FAP-800 sample.

Table S1. Raman vibration modes of phosphate and hydroxyl in the apatitic samples.

Vibration mode	Vibration frequency (cm^{-1})		
	Penel et al., 1997 *	Present work	
		HAP	FAP
PO_4 ν1	965	961	964
PO_4 ν2	432	430	430
	449	450	450
PO_4 ν3	-	1028	-
	1034	1034	1033
	1042	1047	1043
	1053	1057	1053
	1061	1063	1063
	1081	1075	1078
PO_4 ν4	581	578	578
	592	587	587
	608	606	606
	617	616	616
PO_4 ν1	3573-HAP	3573	-
	3537-FAP	-	3537

** Penel, G.; Leroy, G.; Rey, C.; Sombret, B.; Huvenne, J.P.; Bres, E. Infrared and Raman microspectrometry study of fluor- fluor-hydroxy and hydroxy-apatite powders. Journal of Materials Science: Materials in Medicine* ***1997****, 8, 271-276, doi:10.1023/A:1018504126866.*
